# Comparative effectiveness of virtual reality vs. traditional motor games on developmental functioning and motor skills in autistic children spectrum disorder: a cohort study

**DOI:** 10.3389/fped.2026.1913344

**Published:** 2026-08-27

**Authors:** Yinlu Wang, Junjie Liu, Ying Li, Yang Liu, Xiaoshan Dai, Xue Wang, Heming Chen, Zheheng Jia, Mi Zheng

**Affiliations:** 1 Graduate School, Harbin Sport University, Harbin, China; 2 School of Basic Medical Sciences, China Three Gorges University, Yichang, China; 3 College of Science and Technology, China Three Gorges University, Yichang, China; 4 Department of Child Rehabilitation, The Fourth Hospital of Harbin, Harbin, China

**Keywords:** autism spectrum disorder, exergaming, motor skills, physical fitness, preschool children, virtual reality

## Abstract

**Introduction:**

Autism Spectrum Disorder (ASD) is a prevalent neurodevelopmental condition with progressively increasing prevalence, and traditional sports game interventions often face challenges in achieving sustained improvements in both developmental functioning and motor proficiency.

**Methods:**

This prospective cohort study compared the effects of traditional sports game interventions vs. immersive virtual reality (VR) exergaming on developmental functioning and motor skills in autistic children, and evaluated the long-term stability of outcomes. Assessments using the Gesell Developmental Schedules (GDS) and the PREFIT motor test battery were conducted at three time points: pre-intervention, immediately after the 8-week intervention, and 8 weeks after intervention cessation; both protocols were designed by a hospital-based multidisciplinary team.

**Results:**

Immersive VR exergaming demonstrated a trend toward greater improvements in most GDS subdomains and most PREFIT components compared with traditional sports game interventions, with the experimental group showing larger magnitudes of improvement during the intervention period. Scores on some PREFIT subdomains continued to increase during the 8-week follow-up period.

**Discussion:**

Due to the non-randomized design and the absence of a no-treatment control group, these findings should be interpreted as preliminary evidence. The potential advantages of immersive VR exergaming warrant further investigation through more rigorously designed randomized controlled trials.

## 1 Introduction

Autism Spectrum Disorder (ASD) is a core symptoms disorder characterized by persistent deficits in social communication and interaction, alongside restricted/repetitive behaviors (1). Motor impairment is a prevalent comorbidity, affecting 50%–88% of individuals with ASD (2–4). Clinical manifestations include reduced physical fitness (2), gait abnormalities, and impaired fine/gross motor coordination (5). The relative risk of motor impairment in ASD is 22.2-fold higher than in typically developing peers (6), significantly hindering social participation. With ASD prevalence now affecting 1 in 36 children (7), the fastest-growing core symptoms disorder globally, limited therapeutic resources and low treatment adherence necessitate innovative solutions (8, 9). Virtual reality (VR) technology has thus emerged as a promising evidence-based intervention modality.

Virtual reality (VR) is an immersive interactive system integrating multimedia computing, sensing, and simulation technologies that constructs controllable environments through multimodal sensory devices (10). Its core characteristics are interactivity, immersion, and imagination—enable realistic simulation of real-life scenarios (11, 12). By providing safe, controllable, and highly immersive environments, VR establishes ideal training conditions for individuals with ASD (13). Within virtual interactive environments, most patients exhibit heightened engagement,while concurrently reducing comorbid maladaptive behaviors (14, 15). Accumulating evidence confirms that VR-based interventions significantly enhance social skills, emotion recognition/regulation capabilities, and executive function in children/adolescents with ASD (16–20). Leveraging these cognitive characteristics, regular scenario-specific training improves social adaptability in ASD patients (21, 22). Compared to traditional interventions, VR demonstrates lower implementation costs (23), optimized therapeutic efficiency (24), and more sustained treatment effects (25). Although many studies have reported beneficial effects of VR interventions in autistic populations, the evidence is not uniformly positive. For instance, Simões found that while virtual reality immersion effectively rescaled interpersonal distance regulation in typically developing controls, this effect was absent in autistic participants, suggesting that certain social-cognitive processes may not respond to VR manipulation in this population (26).

Motor skills training and physical activity interventions effectively alleviate symptoms in autistic children spectrum disorder (ASD), enhancing emotional and motor development (27), social-communication skills (28), and quality of life (29). Notably, VR-based motor training programs construct immersive multisensory environments that promote participation in collaborative motor tasks through low-risk, high-motivation engagement (30–33). Leveraging the scenario controllability of virtual environments, these programs improve movement accuracy and skill transfer efficiency for complex motor skills (34). Studies confirm that VR-trained ASD children demonstrate significantly superior motor abilities vs. real-environment training, with sustained intervention effects (34–36). Furthermore, VR training facilitates greater motor skill generalization, enabling application of learned skills to daily living (34, 37–39), establishing it as an effective adjunct to traditional motor intervention strategies (17).

However, most existing studies have focused on improving social skills, emotional status, or overall motor skills of autistic children, but exploration of effects at the detailed level of motor skills is limited. Meanwhile, compared with traditional exercise games, the effects of immersive virtual reality exergaming intervention on autistic children remain unclear and warrant further investigation. Based on this, this study conducted a prospective controlled cohort study to observe and compare the effects of traditional exercise training and VR-based exercise training on developmental functioning and motor skills of autistic children, and to evaluate long-term effect sustainability. The Gesell Scale and Physical Fitness Test Battery for Preschool Children (PREFIT) battery motor test were used. We hypothesized that an 8-week VR exergaming intervention would significantly improve development and motor skills in autistic children, and compared with traditional exercise games, its effects and long-term stability would be superior. This study provides evidence-based support for clinical application of VR technology in ASD rehabilitation, promoting technological progress of VR in ASD treatment.

## 2 Methods

### 2.1 Participants

A prospective, non-randomized observational cohort design was used in this study. The subjects were recruited from the pediatric rehabilitation department of a hospital, and a total of 40 autistic children were included (34 males and 6 females, sex ratio 5: 1, consistent with the epidemiological characteristics of the ASD population).

The grouping process is as follows: Before enrollment, the research nurse provided standardized written and oral instructions to all eligible parents of children, detailing the specific content, training duration, equipment differences and costs of traditional sports game therapy and virtual reality-based sports game therapy. On the premise of full knowledge, the grouping was completely based on parental preference: children who chose VR therapy were included in cohort 1 (*n* = 20; there were 17 males and 3 females. Mean age = 56.64 ± 11.27). children who chose traditional therapy were included in cohort 2 (*n* = 20; there were 17 males and 3 females. The average age = 56.49 ± 10.31), Two senior pediatric rehabilitation physicians independently screened medical records to ensure that all included subjects met the inclusion criteria. Because this study is based on the real clinical environment grouping of parents ‘wishes, and the sample size is limited (20 cases in each group), this study is located in exploratory analysis. The baseline comparability between groups is only used as a reference index and is not used to infer no selection bias. All participants included in the analysis in this study completed all 8-week intervention courses and two assessments (T0, T1, T2). The completion rate of intervention was 100%, and there were no cases of withdrawal or loss of follow-up.

### 2.2 Ethical considerations

Inclusion criteria: (1) Met DSM-5 ASD diagnostic criteria. (2) Mild-to-moderate CARS scores (30–37) with task comprehension ability. (3) Aged 3–7 years. (4) Guardian-signed informed consent. Ethical approval was obtained from the institutional review board prior to study commencement. All guardians provided written informed consent in accordance with the Declaration of Helsinki. Principles of participant privacy and data security were strictly maintained.

### 2.3 Assessment process

Before the study begins, professional physicians will diagnose all participating children and conduct CARS assessments to confirm they meet the criteria for grade 1 or grade 2 ASD, and obtain signed informed consent forms. Once consent forms are signed, participants will undergo three assessments: Baseline assessment (T0): Conducted within 48 h before the intervention starts, this evaluates children's developmental functions and motor abilities pre-intervention. Immediate post-intervention assessment (T1): Performed within 48 h after the 8-week intervention ends, this assesses the immediate effects of both interventions. Follow-up assessment (T2): Carried out 8 weeks post-intervention conclusion, this examines the sustained effects of the interventions.

The Gesell Scale and PREFIT battery motor test are the assessment tools. Baseline assessment (T0) data collection ensures group balance in Gesell Development Diagnosis Scale and PREFIT motor test scores, ensuring pre-intervention comparability of key observation indicators. All assessments are conducted by professional evaluators. They record test data accurately and objectively per standardized procedures and tools, completing detailed test record forms.

### 2.4 Tools

The assessment protocol used in this study includes the following testing tools: the Gesell Developmental Schedules (GDS) (40), and the PREFIT battery (41, 42).

The Gesell Developmental Schedules (GDS) (40) are designed based on the “maturity dominance theory” to test children's core symptoms status and are suitable for children aged 0 to 6 years. The scale assesses the following five key domains: gross motor, fine motor, language ability, adaptability, and personal-social behavior. It reflects children's hand-eye coordination and cognitive processing, the nervous system's neuromuscular control, neural integration during fine operations, executive function, the core symptoms maturity in social adaptability, as well as the language center development and communication proficiency.

The PREFIT battery (41, 42) is a field-based assessment tool used to evaluate the physical fitness of preschool children. It includes the following tests: the 20 m shuttle run, the handgrip strength test, the standing long jump, the 4 × 10 m shuttle run, and the single-leg stance. This tool provides a standardized testing method, covers multiple physical fitness domains, can comprehensively evaluate children's motor competence, and has cross-cultural validity, which can be used in different cultural backgrounds. All its test items demonstrate high feasibility and reliability among preschool children (43).

### 2.5 Intervention content

The intervention measures in this study were all routine treatment programs in the department. All intervention plans were independently formulated and implemented by the multidisciplinary team of the hospital according to the standardized treatment path of the department. And complete standard operating manual. The therapists participating in the intervention must complete unified training and pass the operation assessment before implementation to ensure that the intervention procedures, techniques and guidance standards of each group are highly unified. The researchers in this study were not involved in the formulation or implementation of any intervention measures, and were only responsible for blind assessment and data collection to minimize the impact of subjective bias on the intervention.

Immersive Virtual Reality Exergaming: This study uses a non-head-mounted multimedia scene interaction system based on projection mapping. The hardware devices include infrared motion capture sensors, high-definition projection interaction terminals, stereo audio devices, and somatosensory interaction terminals. The system directly images on the ground without any auxiliary equipment, and realizes real-time motion capture through the body 's body movements, such as children 's feet, hands, and various parts of the body. Real-time acquisition of children 's limb displacement, balance posture, and motion trajectory for scene interaction training. The system is equipped with a special children 's rehabilitation training software, which contains more than one hundred and thirty games. It integrates traditional/digital games into one, combines somatosensory games, and promotes exercise through audio-visual stimulation in a multi-scene environment. Operated by a certified therapist and providing real-time guidance (non-automated training). The game content is the same as the traditional somatosensory game intervention. The whole training process was guided by a certified rehabilitation therapist in real time. Therapists are responsible for monitoring children and ensuring their safety; guide children to complete tasks through voice prompts and demonstration actions; adjust task parameters (such as speed, range, difficulty fine-tuning) according to children 's real-time performance; give children positive feedback and encouragement to maintain participation motivation. The VR system itself does not provide automated training, and all task instructions, feedback and progress decisions are manually controlled by the therapist.

Traditional Sports Game Intervention: dominated by the therapist, using conventional children 's rehabilitation equipment to carry out offline sports game training. The single course structure, training module, task content, time allocation, and difficulty progression rules were completely consistent with the VR intervention group. The whole process was guided by the therapist, supervised the training of the children in real time, corrected the irregular movements and password guidance training in time, and provided immediate error correction and targeted guidance.

The intervention principles and curriculum arrangements of the two groups: The training tasks of the two groups are completely consistent, following the principle of easy to difficult, new and old alternation, covering six training modules: balance control, muscle activation, limb coordination, flexible stretching, object manipulation and sensory perception integration. Both groups of children were given a 50 min VR-based sports game intervention or traditional sports game intervention once a day. Each lesson includes the pre-class part, the main part and the after-class stretching. The pre-class part mainly includes greetings and warm-up exercises, which take 10 min. The main part mainly includes balance ability training, muscle strength training, flexibility training, coordination ability training, object control motor skills and sensory perception training, which takes 35 min. Relaxation after class mainly includes stretching exercise and saying goodbye to each other, which takes 5 min. The training difficulty gradient, task update rhythm and movement content structure of the two groups were completely consistent. The training items are shown in Table 1.

**Table 1:** Intervention protocols and content in both groups.

Intervention phase	Training project	
The first stage (1–2 weeks)	Warm-up activities (5 min)	Jogging, arm cross, arm windmill
	Formal movement (20 min)	Stand on one foot, tiptoe, tiptoe jump, knees on the same side, feet jump left and right, feet jump back and forth
	Relaxation training (5 min)	Leg triceps stretch, chest stretch, massage
The second stage (3–4 weeks)	Warm-up activities (5 min)	Jogging, arm cross, arm windmill
	Formal movement (20 min)	Standing on one foot to move things, tiptoe jump, the other hand knees, *in situ* rotation jump, open and close jump, load pull objects
	Relaxation training (5 min)	Leg triceps stretch, chest stretch, massage
The third stage (5–6 weeks)	Warm-up activities (5 min)	Jogging, arm cross, arm windmill
	Formal movement (20 min)	Single foot jump, ipsilateral hand touch foot, open and close jump, fast running, vertical jump, load pull
	Relaxation training (5 min)	Leg triceps stretch, chest stretch, massage
The fourth stage (7–8 weeks)	Warm-up activities (5 min)	Jogging, Arm Cross, Open and Close Jump
	Formal movement (20 min)	Single—foot jump, fast—running, weight—bearing vertical jump, arm crawl, long jump, weight—bearing pull, Mountain Climber
	Relaxation training (5 min)	Leg triceps stretch, chest stretch, massage

Homogeneity control of inter-group intervention: This study strictly controlled the non-experimental confounding variables of the two groups of interventions. The two groups of interventions were completed by the same certified rehabilitation therapist to ensure that children received the same length of therapist contact, one-to-one guidance frequency, immediate error correction feedback, humanistic attention and interaction intensity. The traditional group used the real scene form, and the VR group used the virtual scene interaction form. In addition to the differences in the media presentation, the training task, exercise load, intervention frequency, course of treatment, external attention and intervention novelty experience all remained balanced between groups, excluding the interference of non-experimental factors on the outcome of the intervention. In addition, both groups of therapists used standardized feedback protocols, including positive language enhancements such as “ well done” when completing the action correctly; give demonstration and correction guidance when the action is not standardized; summative feedback was given after each task, and the frequency and content of feedback were consistent between the two groups. The only inter-group difference is the presentation medium.

Both groups received a 2-month intervention (5 times a week, 50 min each time), and the exercise intensity was controlled at 65% −70% of the maximum heart rate. During the training, a wearable heart rate monitoring device (heart rate chest belt) was used to monitor the exercise load in real time to ensure that the intensity was always maintained in the target range. Follow-up was conducted 2 months after the intervention. Participants who only completed ≥90% of the training sessions were included in the analysis to ensure that the intervention process, training content and guidance standards of each group were highly unified. The specific training contents of the two groups are shown in Table 1.

### 2.6 Data analysis

In this study, SPSS 26.0 statistical software was used for data analysis. The test level *α* = 0.05, *P* < 0.05 was considered statistically significant. After Shapiro–Wilk normality test, the measurement data were expressed as mean ± standard deviation, and the independent sample t test was used for comparison between groups. The non-normal distribution was expressed as median (lower quartile, upper quartile), and the Mann–Whitney *U*-test was used for comparison between groups. The linear mixed effect model (LMM) was used to analyze the changes of the two groups of Gesell Development Scale and PREFIT physical fitness test indicators before intervention (T0), after intervention (T1) and follow-up period (T2). The group, time and group × time interaction term were fixed effects, and the subject ID was a random effect. For the indicators with significant interaction effects, simple effect analysis with Bonferroni correction was used for pairwise comparison between groups and within groups. GraphPad Prism 9.0 was used to draw a cluster histogram to display the results.

## 3 Results

### 3.1 Comparison of baseline characteristics between the two groups

The results of baseline balance test between groups before intervention showed that the age, Gesell Developmental Diagnostic Scale and PREFIT physical fitness test of the two groups were similar by independent sample t test and rank sum Mann–Whitney *U*-test analysis. There was no significant difference in all baseline indicators between the two groups (all *P* > 0.05), indicating that the baseline data of the two groups were balanced and comparable, which could eliminate the interference of baseline confounding factors and meet the research conditions for the comparison of follow-up intervention effects. See Table 2 for details.

**Table 2:** Basic information and baseline characteristics of participants.

Indicator name	Cohort 1 M ± SD/M(P25,P75)(mo)	Cohort 2 M ± SD/M(P25,P75)(mo)	T/u	*P*
Age	56.64 ± 11.27	56.49 ± 10.31	0.04	0.965
Gesell development diagnosis scale				
Adaptability	55.95 ± 17.39	56.35 ± 21.69	0.064	0.949
Gross motor	58.50 ± 16.14	59.25 ± 19.78	0.131	0.896
Fine motor skills	58.30 ± 18.39	58.90 ± 20.51	0.097	0.923
Language	50.10 ± 16.70	51.00 ± 21.35	0.148	0.883
Personal-Social	57.15 ± 17.87	57.40 ± 20.9	0.041	0.968
PREFIT battery				
Grip	4.10 (3.55, 4.78)	3.95 (3.73, 5.00)	197.00	0.935
Standing jump	52.92 ± 9.97	53.80 ± 9.95	0.282	0.780
20 m shuttle run Test	9.00 ± 2.94	9.50 ± 2.72	0.558	0.580
4 × 10 m shuttle run	33.29 (24.52, 42.04)	29.75 (25.30, 33.98)	180.00	0.589
One-Leg Stance	7.09 (5.01, 10.64)	6.84 (5.92, 7.80)	194.00	0.871

### 3.2 Gesell developmental schedules

#### 3.2.1 Comparison of adaptability scores between the two groups at different time points using a mixed model

**Figure 1:**
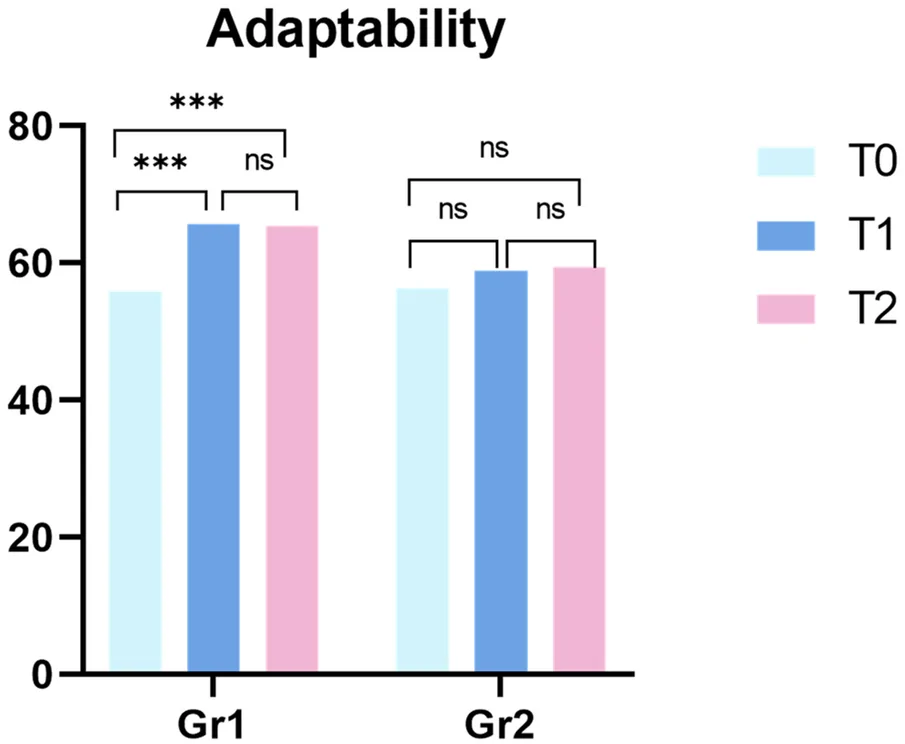
Comparison of cohort 1 and cohort 2 at T0, T1 and T2 in adaptability. *the accepted significance is *p* < .05, ** the accepted significance is *p* < .01, ***the accepted significance is *p* < .001.

The linear mixed model was used to analyze the adaptability scores of the two groups of autistic children T0, T1 and T2. The higher the score of the index, the better the adaptability of the children. The results of fixed effect test showed that the main effect of time was significant (F (2,76) = 28.944, *p* < 0.001), indicating that the adaptability increased significantly with time, and autistic children showed different degrees of improvement under both intervention conditions. The main effect of the group was not significant (F (1,38) = 0.459, *p* = 0.502), suggesting that there was no significant difference in the overall level of adaptability between the two groups; the interaction effect of time × group was extremely significant (F (2, 76) = 8.825, *p* < 0.001, partial *η*^2^ = 0.189), indicating that there was a significant difference in the longitudinal change trajectory of the adaptive score between the two groups, that is, the change patterns associated with different intervention methods were different. See Table 3 and Figure 1 for details.

**Table 3:** Tests of fixed effects for *adaptability* scores across two groups and three time points.

Time	Cohort 1	Cohort 2	Tests of Fixed Effects		
	M ± SD	M ± SD	F	*P*	Partial *η*^2^
Before intervention	55.95 ± 4.37	56.35 ± 4.37			
After intervention	65.65 ± 4.37	58.90 ± 4.37			
Follow-up	65.35 ± 4.37	59.35 ± 4.37			
Time main effect			28.944	<0.001	
Group main effect			0.459	0.502	
Time × Group effect			8.825	0.001	0.189

Post hoc pairwise comparison showed that the core difference between the two groups was mainly reflected in the intervention stage (T0 → T1): the adaptability score of Cohort 1 was significantly improved (*Δ* =  + 9.70, *p* < 0.001), and the improvement was about 3.8 times that of Cohort 2 (*Δ* =  + 2.55, *p* = 0.173), while the improvement of Cohort 2 did not reach a significant level. After entering the follow-up period (T1 → T2), the Cohort 1 score remained stable (*Δ* = −0.30, *p* = 1.000), and there was no significant fluctuation in Cohort 2 (*Δ* =  + 0.45, *p* = 1.000).

On the whole, neither of the two intervention models had a negative impact on autistic children. Immersive Virtual Reality Exergaming has a time-dependent relationship with the significant improvement of adaptability score during the intervention period, and this high change shows good stability during the follow-up period; the improvement of adaptability under the condition of Traditional Sports Game Intervention is relatively limited, but there is no regression. This result suggests that Immersive Virtual Reality Exergaming may provide an effective promotion path for autistic children adaptation. However, limited by non-randomized design and the lack of treatment-free control group, the above findings should be used as an exploratory reminder that the potential advantages of Immersive Virtual Reality Exergaming need to be verified by more rigorously designed randomized controlled trials. The effect of Traditional Sports Game Intervention is not significant.

#### 3.2.2 Comparison of gross motor scores between the two groups at different time points using a mixed model

**Figure 2:**
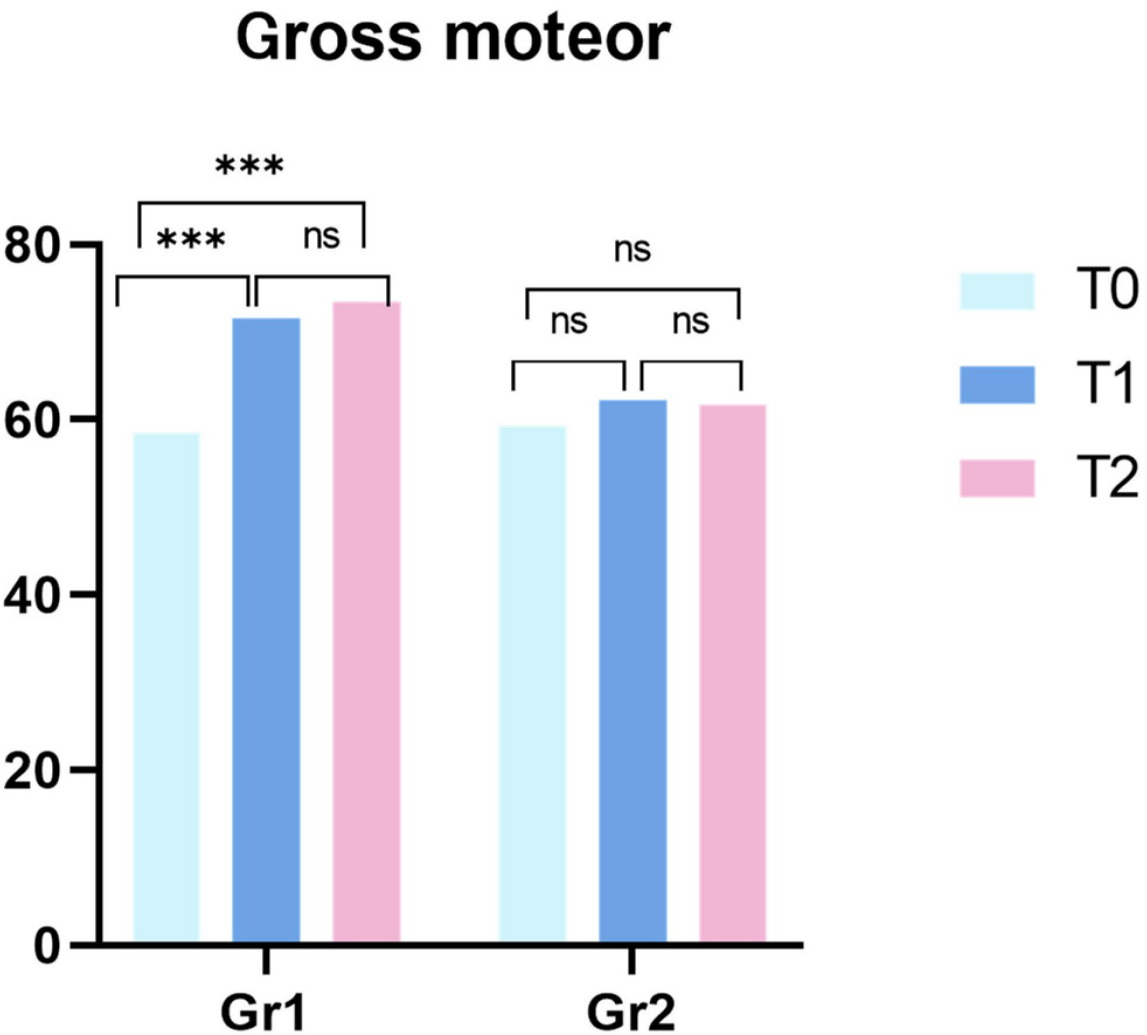
Comparison of Cohort 1 and Cohort 2 at T0, T1 and T2 in Gross motor. *the accepted significance is *p* < .05, ** the accepted significance is *p* < .01, ***the accepted significance is *p* < .001.

The linear mixed model was used to analyze the gross motor scores of the two groups of autistic children T0, T1 and T2. The higher the score of this index, the better the gross motor ability of children. The results of fixed effect test showed that the main effect of time was significant (F (1.593,60.524) = 50.270, *p* < 0.001), indicating that gross motor ability increased significantly with time, and autistic children showed different degrees of improvement under both intervention conditions. The main effect of the group was not significant (F (1,38) = 1.341, *p* = 0.254), suggesting that there was no significant difference in the overall level of gross motor between the two groups; the interaction effect of time × group was significant (F (1.593, 60.524) = 23.492, *p* < 0.001), indicating that there were substantial differences in the longitudinal change trajectory of gross motor scores between the two groups, that is, the change patterns associated with different intervention methods were different. See Table 4 and Figure 2 for details.

**Table 4:** Tests of fixed effects for gross motor scores across two groups and three time points.

Time	Cohort 1	Cohort 2	Tests of Fixed Effects		
	M ± SD	M ± SD	F	*P*	Partial η^2^
Before intervention	58.50 ± 4.21	59.25 ± 4.21			
After intervention	71.60 ± 4.21	62.25 ± 4.21			
Follow-up	73.40 ± 4.21	61.70 ± 4.21			
Time main effect			50.270	<0.001	
Group main effect			1.341	0.254	
Time × Group effect			23.492	<0.001	0.382

Post hoc pairwise comparison showed that the core difference between the two groups was mainly reflected in the intervention stage (T0 → T1): the gross motor score of Cohort 1 was significantly increased (*Δ* =  + 13.10, *p* < 0.001), and the improvement was about 5.1 times that of Cohort 2 (*Δ* =  + 2.55 *p* = 0.093), while the improvement of Cohort 2 did not reach a significant level. After entering the follow-up period (T1 → T2), the Cohort 1 score continued to increase slightly (*Δ* =  + 1.80, *p* = 0.573), and the Cohort 2 score decreased slightly.

On the whole, neither of the two intervention models had a negative impact on autistic children. Immersive Virtual Reality Exergaming has a time-dependent relationship with the significant improvement of gross motor ability during the intervention period, and this change shows good stability during the follow-up period; the improvement of gross motor ability under the condition of Traditional Sports Game Intervention is relatively limited. This result suggests that Immersive Virtual Reality Exergaming may provide an effective way to promote the development of gross motor development in autistic children. However, limited by non-randomized design and lack of treatment-free control group, the above findings should be used as exploratory tips. The potential advantages of Immersive Virtual Reality Exergaming need to be verified by more rigorously designed randomized controlled trials; the effect of Traditional Sports Game Intervention is not significant.

#### 3.2.3 Comparison of fine motor scores between the two groups at different time points using a mixed model

The linear mixed model was used to analyze the Fine Motor scores of the two groups of autistic children T0, T1 and T2. The higher the score of this index, the better the fine motor ability of children. The results of fixed effect test showed that the main effect of time was significant (F (2, 76) = 4.340, *p* = 0.016), indicating that the fine motor ability increased significantly with time, and autistic children showed a certain degree of improvement trend under the two intervention conditions. The main effect of the group was not significant (F (1,38) = 0.006, *p* = 0.940), suggesting that there was no significant difference in the overall level of fine motor between the two groups; the interaction effect of time × group was not significant (F (2, 76) = 0.707, *p* = 0.496, partial *η*^2^ = 0.018), indicating that the longitudinal change trajectory of fine motor scores in the two groups was basically parallel, and the intervention type did not have a substantial adjustment effect on the change trend. Because the interaction effect was not significant, according to the statistical specification, this study did not compare the time points in the group. See Table 5 for details.

**Table 5:** Tests of fixed effects for fine motor scores across two groups and three time points.

Time	Cohort 1	Cohort 2	Tests of Fixed Effects		
	M ± SD	M ± SD	F	*P*	Partial η²
before intervention	58.30 ± 4.25	58.90 ± 4.25			
after intervention	61.30 ± 4.25	60.30 ± 4.25			
follow-up	59.75 ± 4.25	58.80 ± 4.25			
time main effect			4.340	0.016	
Group main effect			0.006	0.940	
time × Group effect			0.707	0.496	

On the whole, the fine motor function of the two groups showed a slightly parallel upward trend. The improvement effect of VR sports games on the fine motor ability of autistic children is basically equivalent to that of traditional sports games. Immersive Virtual Reality Exergaming does not show an additional gain effect in this sample. The results suggest that in the field of fine motor, there may be no essential difference between the effects of the two interventions, and the improvement may be mainly due to time factors (such as natural development or practice effects) rather than specific interventions.

#### 3.2.4 Comparison of language scores between the two groups at different time points using a mixed model

**Figure 3:**
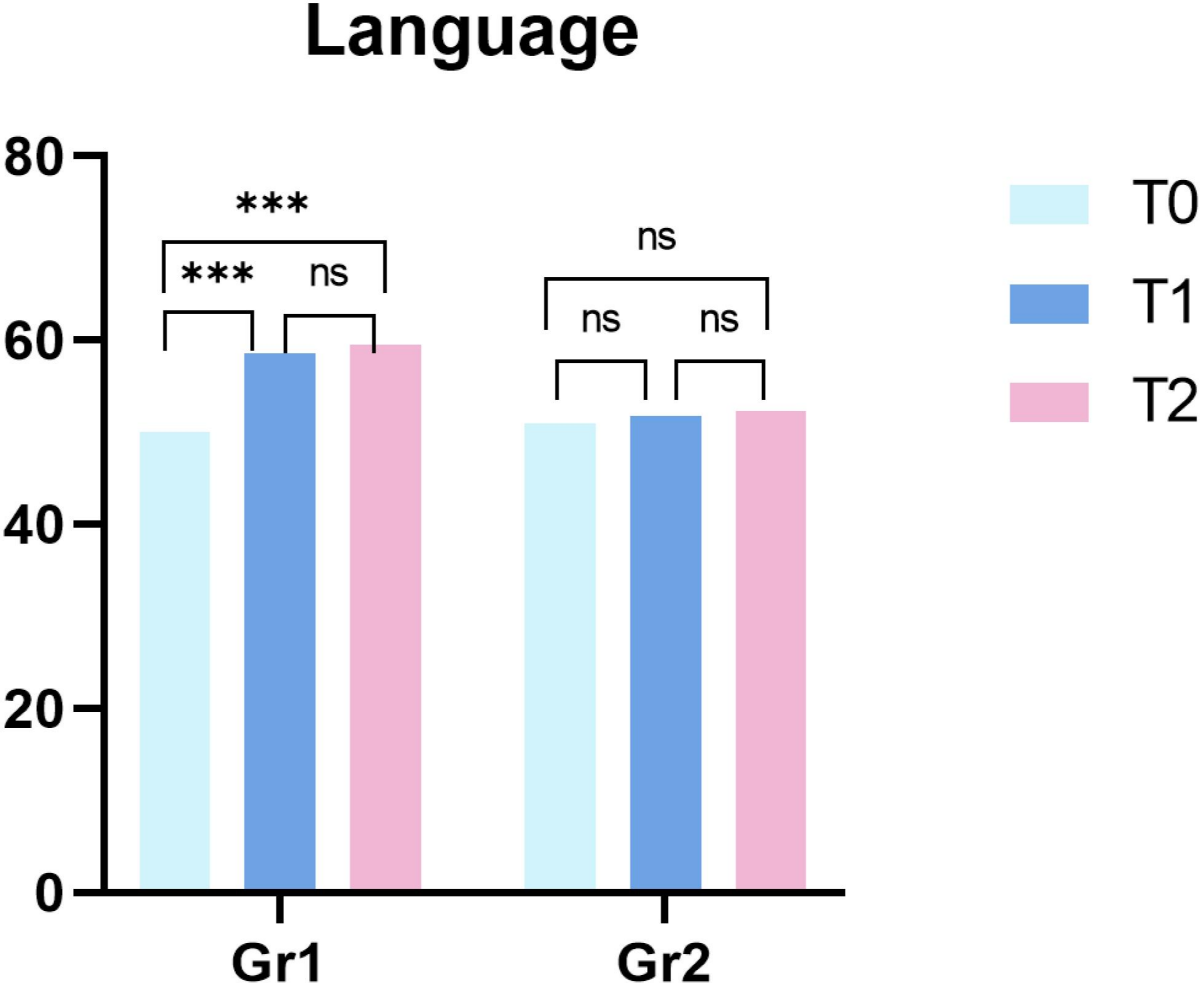
Comparison of cohort 1 and cohort 2 at T0, T1 and T2 in language ability. *the accepted significance is *p* < .05, ** the accepted significance is *p* < .01, ***the accepted significance is *p* < .001.

The linear mixed model was used to analyze the language scores of the two groups of autistic children T0, T1 and T2. The higher the score of the index, the better the children 's speech development ability. The results of fixed effect test showed that the main effect of time was significant (F (2,76) = 19.818, *p* < 0.001), indicating that language ability increased significantly with time, and autistic children showed different degrees of improvement under both intervention conditions. The main effect of the group was not significant (F (1,38) = 0.549, *p* = 0.463), suggesting that there was no significant difference in the overall language level between the two groups; the interaction effect of time × group was significant (F (2, 76) = 12.366, *p* < 0.001), indicating that there were substantial differences in the longitudinal change trajectory of language scores between the two groups, that is, the change patterns associated with different intervention methods were different. See Table 6 and Figure 3 for details.

**Table 6:** Tests of fixed effects for language scores across two groups and three time points.

Time	Cohort 1	Cohort 2	Tests of Fixed Effects		
	M ± SD	M ± SD	F	*P*	Partial η^2^
Before intervention	50.10 ± 4.28	51.00 ± 4.28			
After intervention	58.65 ± 4.28	51.75 ± 4.28			
Follow-up	59.55 ± 4.28	52.30 ± 4.28			
Time main effect			19.818	<0.001	
Group main effect			0.549	0.463	
Time × Group effect			12.366	<0.001	0.245

Post hoc pairwise comparison (Bonferroni correction) showed that the core difference between the two groups was mainly reflected in the intervention phase (T0 → T1): the language score of Cohort 1 increased significantly (*Δ* =  + 8.55, *p* < .001), while the change of Cohort 2 did not reach a significant level (*Δ* =  + 0.75, *p* = 1.000). After entering the follow-up period (T1 → T2), the score of Cohort 1 continued to increase slightly (*Δ* =  + 0.90, *p* = 1.000), and Cohort 2 also showed a slight increase (*Δ* =  + 0.55, *p* = 1.000). The changes in the two groups did not reach a statistically significant level.

On the whole, neither of the two intervention models had a negative impact on autistic children, and all autistic children showed different degrees of positive development trajectories in language ability. Immersive Virtual Reality Exergaming has a time-dependent relationship with the significant improvement of language scores during the intervention period, and this high change shows good stability during the follow-up period; the improvement of language ability under the condition of Traditional Sports Game Intervention is relatively limited, but there is no regression. This result suggests that Immersive Virtual Reality Exergaming may provide an effective way to promote language ability in autistic children. However, limited by non-randomized design and lack of treatment-free control group, the above findings should be used as exploratory tips. The potential advantages of Immersive Virtual Reality Exergaming need to be verified by more rigorously designed randomized controlled trials and larger sample studies; although no significant changes were observed in Traditional Sports Game Intervention in this study, its auxiliary value in comprehensive rehabilitation cannot be ruled out.

#### 3.2.5 Comparison of personal-social scores between the two groups at different time points using a mixed model

**Figure 4:**
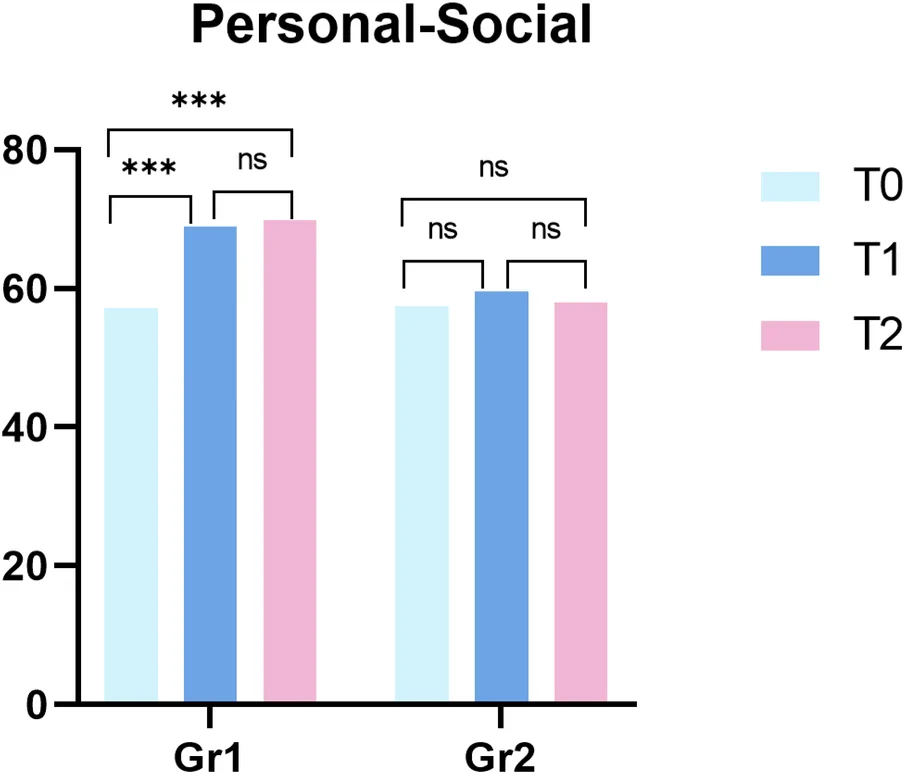
Comparison of cohort 1 and cohort 2 at T0, T1 and T2 in personal-social. *the accepted significance is *p* < .05, ** the accepted significance is *p* < .01, ***the accepted significance is *p* < .001.

The linear mixed model was used to analyze the personal social scores of the two groups of autistic children T0, T1 and T2. The higher the score of the index, the better the children 's personal social ability. The results of fixed effect test showed that the main effect of time was significant (F (2, 76) = 46.541, *p* < 0.001), indicating that social ability increased significantly with time, and autistic children showed different degrees of improvement under the two intervention conditions. The main effect of the group was not significant (F (1,38) = 1.406, *p* = 0.243), suggesting that there was no significant difference in the overall level of social interaction between the two groups; the interaction effect of time × group was significant (F (2, 76) = 30.883, *p* < 0.001), indicating that there was a substantial difference in the longitudinal change trajectory of social scores between the two groups, that is, the change patterns associated with different intervention methods were different. See Table 7 and Figure 4 for details.

**Table 7:** Tests of fixed effects for personal social behavior scores across two groups and three time points.

Time	Cohort 1	Cohort 2	Repeated measurement inspection		
	M ± SD	M ± SD	F	*P*	Partial η^2^
Before intervention	57.15 ± 4.24	57.40 ± 4.24			
After intervention	68.95 ± 4.24	59.60 ± 4.24			
Follow-up	69.90 ± 4.24	57.95 ± 4.24			
Time main effect			46.541	<0.001	
Group main effect			1.406	0.243	
Time × Group effect			30.883	<0.001	0.448

Post-hoc pairwise comparison (Bonferroni correction) showed that the core difference in the trajectory of the two groups was mainly reflected in the intervention stage (T0 → T1): the social score of Cohort 1 increased significantly (*Δ* =  + 11.80, *p* < 0.001), and the improvement was about 5.4 times that of Cohort 2 (*Δ* =  + 2.20, *p* = 0.183), while the improvement of Cohort 2 did not reach a significant level. After entering the follow-up period (T1 → T2), the Cohort 1 score continued to increase slightly (*Δ* =  + 0.95, *p* = 1.000), and Cohort 2 showed a slight decrease (*Δ* = −1.65, *p* = 0.473).

On the whole, neither of the two intervention models had a significant negative impact on autistic children. Immersive Virtual Reality Exergaming has a time-dependent relationship with the significant increase in social scores during the intervention period, and this change shows good stability during the follow-up period; the improvement of social ability under the condition of Traditional Sports Game Intervention was relatively limited, and the follow-up period fell slightly but did not reach a significant level. This result suggests that Immersive Virtual Reality Exergaming may provide an effective promotion path for autistic children social competence. However, limited by non-randomized design and lack of treatment-free control group, the above trend is only indicative, which is not enough to infer that Immersive Virtual Reality Exergaming has a lasting clinical effect. The potential advantages of Immersive Virtual Reality Exergaming need to be verified by more rigorously designed randomized controlled trials. Traditional Sports Game Intervention did not observe significant changes in this study, but its auxiliary value in comprehensive rehabilitation cannot be ruled out.

### 3.3 PREFIT battery motor test

#### 3.3.1 Comparison of grip scores between the two groups at different time points using a mixed model

The linear mixed model was used to analyze the Hand Grip scores of the two groups of autistic children T0, T1 and T2. The index evaluates the upper limb strength of children. The higher the score, the better the upper limb strength of children. The results of fixed effect test showed that the main effect of time was significant (F (2, 76) = 17.927, *p* < 0.001), indicating that grip strength increased significantly with time, and autistic children showed a certain degree of improvement trend under both intervention conditions. The main effect of the group was not significant (F (1,38) = 0.939, *p* = 0.339), suggesting that there was no significant difference in the overall level of grip strength between the two groups; the interaction effect of time × group was not significant (F (2, 76) = 1.400, *p* = 0.253), indicating that the longitudinal change trajectory of grip strength in the two groups was basically parallel, and the intervention type did not have a substantive adjustment effect on the change trend. Because the interaction effect was not significant, according to the statistical specification, this study did not compare the time points in the group. See Table 8 for details.

**Table 8:** Tests of fixed effects for the grip scores across two groups and three time points.

Time	Cohort 1	Cohort 2	Tests of Fixed Effects		
	M ± SD(kg)	M ± SD(kg)	F	*P*	Partial η^2^
Before intervention	4.25 ± 0.216	4.13 ± 0.216			
After intervention	4.60 ± 0.216	4.33 ± 0.216			
Follow-up	4.96 ± 0.216	4.53 ± 0.216			
Time main effect			17.927	<0.001	
Group main effect			0.939	0.339	
Time × Group effect			1.400	0.253	0.036

On the whole, the grip function of the two groups showed a roughly parallel upward trend. The improvement effect of the two intervention methods on the upper limb strength of autistic children was basically equivalent, and Immersive Virtual Reality Exergaming did not show additional gain effect in this sample. The results suggest that there may be no essential difference in the role of the two interventions in the field of upper limb strength, and the improvement may be mainly due to time factors rather than specific interventions.

#### 3.3.2 Comparison of standing jump scores between the two groups at different time points using a mixed model

**Figure 5:**
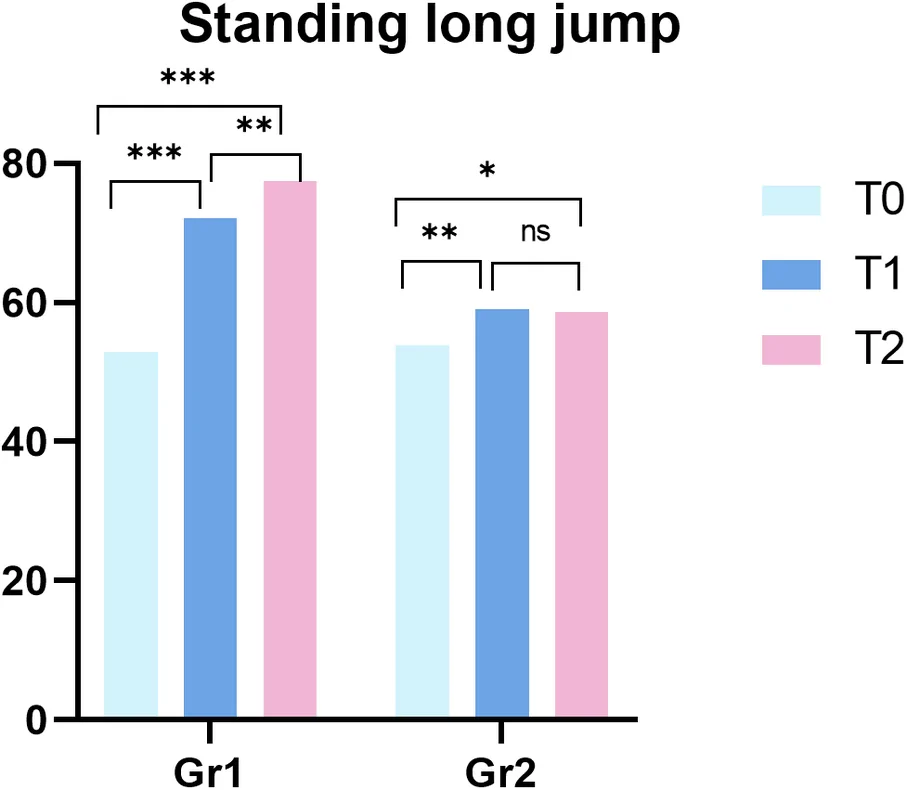
Comparison of cohort 1 and cohort 2 at T0, T1 and T2 in standing long jump. *the accepted significance is *p* < .05, ** the accepted significance is *p* < .01, ***the accepted significance is *p* < .001.

The linear mixed model was used to analyze the Standing Jump scores of the two groups of autistic children T0, T1 and T2. It is worth noting that the Standing Jump evaluates the lower limb explosive force of children. The index is the jumping distance, and the higher the score, the better the lower limb explosive force. The results of fixed effect test showed that the main effect of time was significant (F (2,76) = 97.697, *p* < 0.001), indicating that the standing long jump ability increased significantly with time, and autistic children showed different degrees of improvement under both intervention conditions. The main effect of the group was significant (F (1,38) = 10.145, *p* = 0.003), suggesting that there was a significant difference between the two groups in the overall level of standing long jump; the interaction effect of time × group was extremely significant (F (2, 76) = 40.669, *p* < 0.001, partial *η*^2^ = 0.517), indicating that there was a significant difference in the longitudinal change trajectory of the standing long jump scores between the two groups, that is, the change patterns associated with different intervention methods were different. See Table 9 and Figure 5 for details.

**Table 9:** Tests of fixed effects for the standing long jump scores across two groups and three time points.

Time	Cohort 1	Cohort 2	Tests of Fixed Effects		
	M ± SD (cm)	M ± SD (cm)	F	*P*	Partial η^2^
Before intervention	52.92 ± 2.48	53.80 ± 2.48			
After intervention	72.14 ± 2.48	59.04 ± 2.48			
Follow-up	77.48 ± 2.48	58.63 ± 2.48			
Time main effect			97.697	<0.001	
Group main effect			10.145	0.003	
Time × Group effect			40.669	<0.001	0.517

Post hoc pairwise comparison showed that the core difference between the two groups was mainly reflected in the intervention stage (T0 → T1): the score of Cohort 1 standing long jump was significantly improved (*Δ* =  + 19.22, *p* < 0.001), and the improvement was about 3.7 times that of Cohort 2 (*Δ* =  + 5.24, *p* = 0.005). After entering the follow-up period (T1 → T2), the Cohort 1 score continued to increase significantly (*Δ* =  + 5.35, *p* = 0.004), indicating that Immersive Virtual Reality Exergaming showed a continuous cumulative trend during the follow-up period; the Cohort 2 score was basically flat (*Δ* = −0.41, *p* = 1.000).

On the whole, both intervention models have a positive impact on the lower limb explosive force of autistic children, but Immersive Virtual Reality Exergaming has a greater improvement during the intervention period, and the follow-up period shows a continuous upward trend; although traditional sports games can also bring initial improvement, their effects tend to be stable after the intervention, failing to produce additional cumulative gains. This result suggests that Immersive Virtual Reality Exergaming may provide an effective promotion path for autistic children standing long jump ability. However, limited by non-randomized design and the lack of treatment-free control group, the continuous upward trend of Cohort 1 during the follow-up period may also be partially affected by the practice effect or statistical regression. The above findings should be used as exploratory tips. The potential advantages of Immersive Virtual Reality Exergaming need to be verified by more rigorously designed randomized controlled trials.

#### 3.3.3 Comparison of 20 m shuttle run test scores between the two groups at different time points using a mixed model

**Figure 6:**
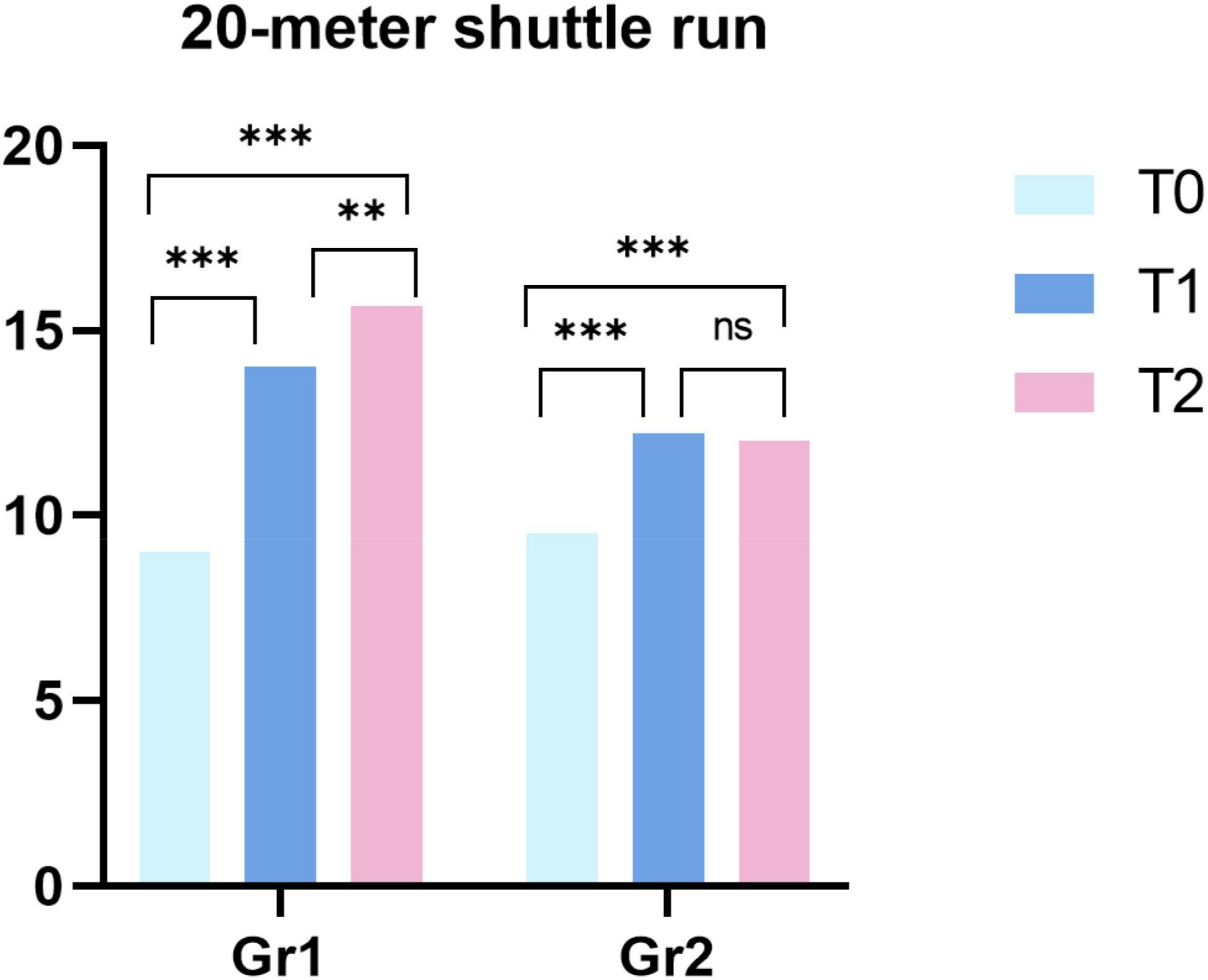
Comparison of cohort 1 and cohort 2 at T0, T1 and T2 in 20 m shuttle run test. *the accepted significance is *p* < .05, ** the accepted significance is *p* < .01, ***the accepted significance is *p* < .001.

The linear mixed model was used to analyze the 20 m shuttle run scores of the two groups of autistic children T0, T1 and T2. It should be noted that the cardiopulmonary endurance of children assessed by the project is measured by the number of laps completed. The more laps, the better cardiopulmonary endurance. The results of fixed effect test showed that the main effect of time was significant (F (2, 76) = 89.013, *p* < 0.001), indicating that the 20 m shuttle running ability increased significantly with time, and autistic children showed different degrees of improvement under the two intervention conditions. The main effect of the group was not significant (F (1,38) = 3.417, *p* = 0.072), suggesting that there was no significant difference between the two groups at the overall level; the interaction effect of time × group was extremely significant (F (2,76) = 15.908, *p* < 0.001, partial *η*^2^ = 0.295), indicating that there was a significant difference in the longitudinal change trajectory of the 20 m shuttle run score between the two groups, that is, the change patterns associated with different intervention methods were different. See Table 10 and Figure 6 for details.

**Table 10:** Tests of fixed effects for the 20 m shuttle run scores across two groups and three time points.

Time	Cohort 1	Cohort 2	Tests of Fixed Effects		
	M ± SD (Laps)	M ± SD (Laps)	F	*P*	Partial η^2^
Before intervention	9.00 ± 0.70	9.50 ± 0.70			
After intervention	14.00 ± 0.70	12.20 ± 0.70			
Follow-up	15.65 ± 0.70	12.00 ± 0.70			
Time main effect			89.013	<0.001	
Group main effect			3.417	0.072	
Time × Group effect			15.908	<0.001	0.295

Post-hoc pairwise comparison showed that the core difference in the change trajectory of the two groups was mainly reflected in the intervention phase (T0 → T1): the Cohort 1 score was significantly increased (*Δ* = +5.00, *p* < 0.001), and the improvement was about 1.85 times that of Cohort 2 (*Δ* = +2.70, *p* < 0.001). Both groups improved after the intervention compared with the baseline. After entering the follow-up period (T1 → T2), the Cohort 1 score continued to increase significantly (*Δ* = +1.65, *p* = 0.007), suggesting that Immersive Virtual Reality Exergaming showed a trend of continuous accumulation during the follow-up period. The Cohort 2 score was basically flat (*Δ* = −0.20, *p* = 1.000).

On the whole, both intervention modes have a positive impact on the cardiorespiratory endurance of autistic children, but Immersive Virtual Reality Exergaming has a greater improvement during the intervention period, and the intervention effect continues to accumulate and steadily increase during the follow-up period. Traditional Sports Game Intervention has a significant improvement, but the effect size is small, and there is no continuous gain during the follow-up period. This result suggests that Immersive Virtual Reality Exergaming may provide an effective promotion path for autistic children 20 m shuttle running ability. However, due to the non-randomized design and the lack of treatment-free control group, the continuous upward trend of Cohort 1 during the follow-up period may also be partially affected by the practice effect or statistical regression. The above findings should be used as exploratory tips. The potential advantages of Immersive Virtual Reality Exergaming need to be verified by more rigorously designed randomized controlled trials.

#### 3.3.4 Comparison of 4 × 10 m shuttle run scores between the two groups at different time points using a mixed model

**Figure 7:**
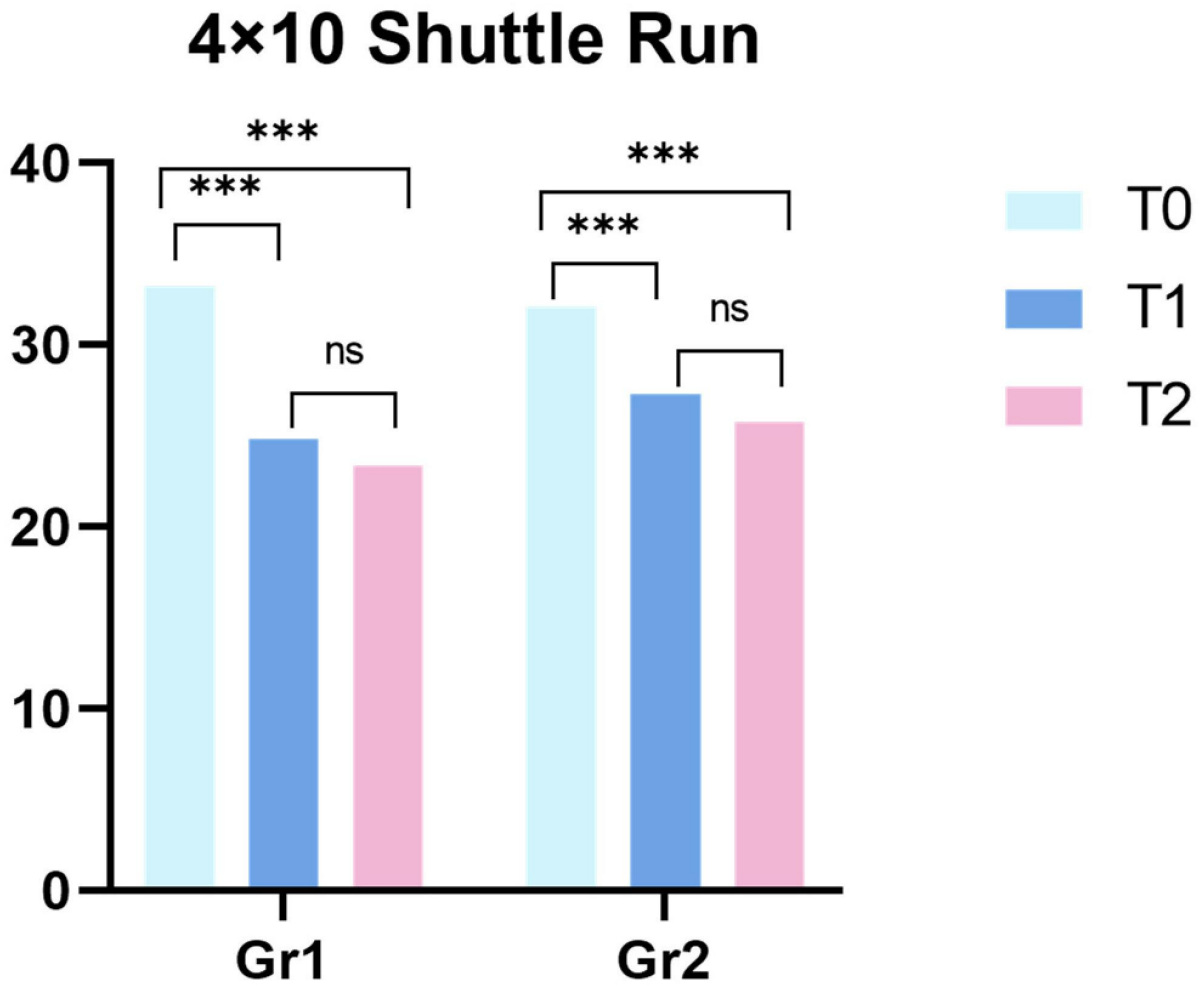
Comparison of cohort 1 and cohort 2 at T0, T1 and T2 in 4 × 10 m shuttle run. *the accepted significance is *p* < .05, ** the accepted significance is *p* < .01, ***the accepted significance is *p* < .001.

The linear mixed model was used to analyze the completion time of the 4 × 10 m shuttle run of the two groups of autistic children T0, T1 and T2. The index evaluates the speed and sensitivity of children. The shorter the completion time, the better the speed and sensitivity. The results of fixed effect test showed that the main effect of time was significant (F (2, 76) = 101.004, *p* < 0.001), indicating that the completion time of 4 × 10 m shuttle run was significantly shortened with time, and autistic children showed different degrees of improvement under the two intervention conditions. The main effect of the group was not significant (F (1,38) = 0.194, *p* = 0.662), suggesting that there was no significant difference in the overall level between the two groups; the interaction effect of time × group was significant (F (2, 76) = 5.771, *p* = 0.005, partial *η*^2^ = 0.132), indicating that there was a substantial difference in the longitudinal change trajectory of the completion time between the two groups, that is, the change patterns associated with different intervention methods were different. See Table 11 and Figure 7 for details.

**Table 11:** Tests of fixed effects for the 4 × 10 m shuttle run scores across two groups and three time points.

Time	Cohort 1	Cohort 2	Tests of Fixed Effects		
	M ± SD (s)	M ± SD (s)	F	*P*	Partial η^2^
Before intervention	33.21 ± 2.13	32.12 ± 2.13			
After intervention	24.82 ± 2.13	27.39 ± 2.13			
Follow-up	23.36 ± 2.13	25.74 ± 2.13			
Time main effect			101.004	<0.001	
Group main effect			0.194	0.662	
Time × Group effect			5.771	0.007	0.132

Post hoc pairwise comparison showed that the core difference in the change trajectory between the two groups was mainly reflected in the intervention phase (T0 → T1): the completion time of Cohort 1 was significantly shortened (*Δ* = −8.39, *p* < 0.001), and the improvement was about 1.77 times that of Cohort 2 (*Δ* = −4.73, *p* < 0.001). Both groups improved after the intervention compared with the baseline. After entering the follow-up period (T1 → T2), the completion time of Cohort 1 continued to be shortened (*Δ* = −1.46, *p* = 0.280), and Cohort 2 was also slightly shortened (*Δ* = −1.65, *p* = 0.176). It should be noted that the absolute value of the change in the follow-up period of Cohort 2 is slightly larger than that of Cohort 1, but the difference is not statistically significant, which may reflect that there is a larger numerical cyclotron space under the condition of lower initial level, and should not be interpreted as the cumulative effect of Traditional Sports Game Intervention is better.

On the whole, both intervention modes have a positive impact on the speed and agility of autistic children, but Immersive Virtual Reality Exergaming has a greater improvement during the intervention period. During the follow-up period, both groups tended to stabilize the platform, and the high-position advantage of Immersive Virtual Reality Exergaming was successfully locked. This result suggests that Immersive Virtual Reality Exergaming may provide an effective promotion path for autistic children 4 × 10 m shuttle running ability. However, limited by non-randomized design and the lack of treatment-free control group, the above findings should be used as exploratory tips. The potential advantages of Immersive Virtual Reality Exergaming still need to be verified by more rigorously designed randomized controlled trials.

#### 3.3.5 Comparison of one leg stance scores between the two groups at different time points using a mixed model

**Figure 8:**
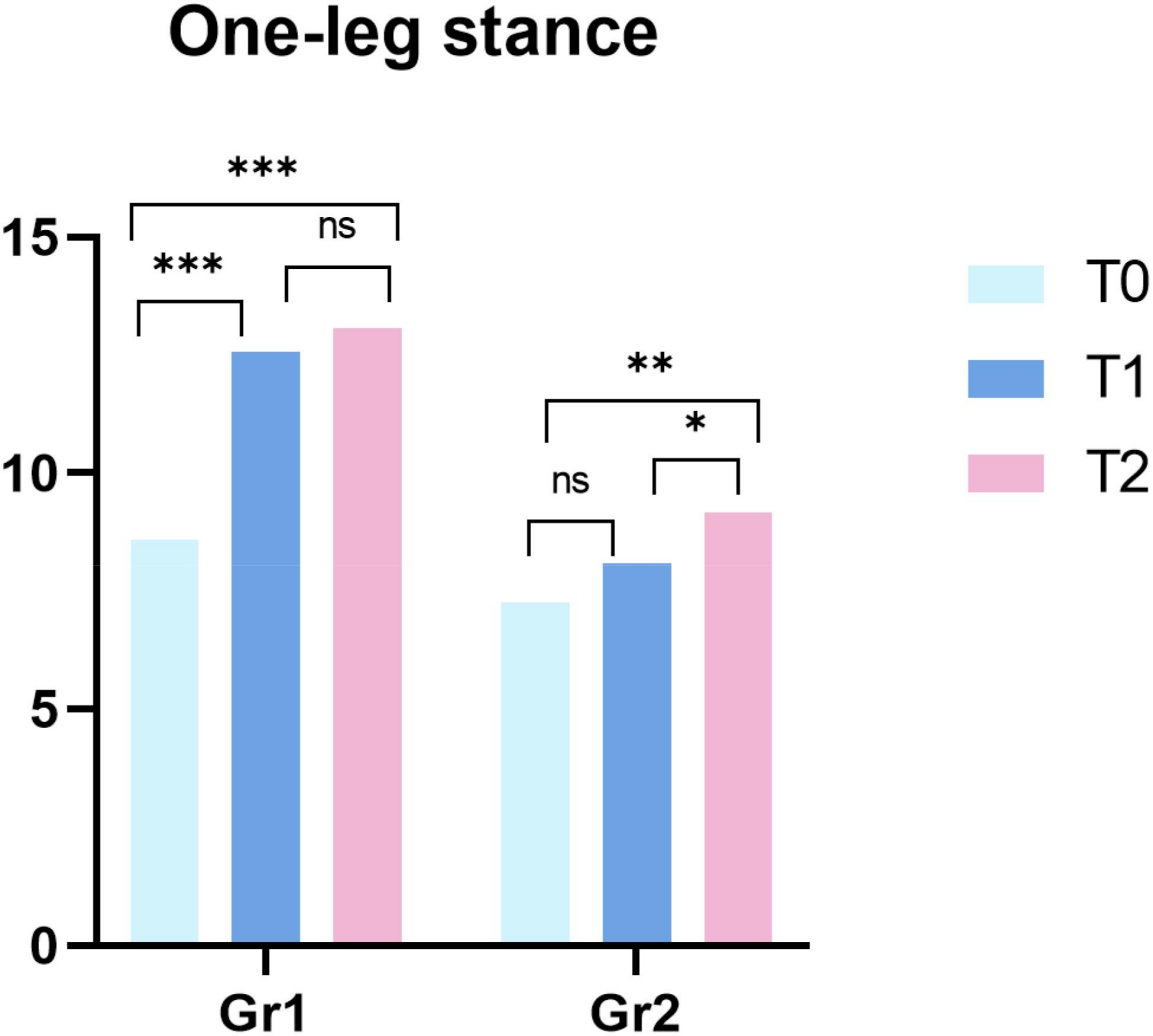
Comparison of cohort 1 and cohort 2 at T0, T1 and T2 in one-leg stance. *the accepted significance is *p* < .05, ** the accepted significance is *p* < .01, ***the accepted significance is *p* < .001.

The linear mixed model was used to analyze the One Leg Stance retention time of the two groups of autistic children T0, T1 and T2. The index evaluates the balance and lower limb static control ability of children. The longer the time, the better the balance and lower limb static control ability. The results of fixed effect test showed that the main effect of time was significant (F (2,76) = 60.539, *p* < 0.001), indicating that the retention time of one-legged standing was significantly prolonged with time, and autistic children showed different degrees of improvement under both intervention conditions. The main effect of the group was significant (F (1,38) = 4.856, *p* = 0.034), suggesting that there was a significant difference between the two groups in the overall level of standing on one foot; the time × group interaction effect was extremely significant (F (2,76) = 15.270, *p* < 0.001, partial *η*2 = 0.287), indicating that there was a significant difference in the longitudinal change trajectory of one-legged standing time between the two groups, that is, the change patterns associated with different intervention methods were different. See Table 12 and Figure 8 for details.

**Table 12:** Tests of fixed effects for One leg stance scores across two groups and three time points.

Time	Cohort 1	Cohort 2	Tests of Fixed Effects		
	M ± SD (s)	M ± SD (s)	F	*P*	Partial η^2^
Before intervention	8.60 ± 1.07	7.26 ± 1.07			
After intervention	12.56 ± 1.07	8.10 ± 1.07			
Follow-up	13.07 ± 1.07	9.17 ± 1.07			
Time main effect			60.539	<0.001	
Group main effect			4.856	0.034	
Time × Group effect			15.270	<0.001	0.287

Post hoc pairwise comparison showed that the core difference between the two groups was mainly reflected in the intervention phase (T0 → T1): the retention time of Cohort 1 was significantly prolonged (*Δ* = +3.96, *p* < 0.001), and the improvement was about 4.7 times that of Cohort 2 (*Δ* = +0.84, *p* = 0.161), while the improvement of Cohort 2 did not reach a significant level. After entering the follow-up period (T1 → T2), the retention time of Cohort 1 continued to be slightly prolonged (*Δ* = +0.51, *p* = 0.735), and Cohort 2 was also prolonged (*Δ* = +1.07, *p* = 0.735). It should be noted that the absolute value of the change in the follow-up period of Cohort 2 is slightly larger than that of Cohort 1, but the difference is not statistically significant, which may reflect that there is a larger numerical cyclotron space under the condition of lower initial level, and should not be interpreted as the cumulative effect of Traditional Sports Game Intervention is better.

On the whole, Immersive Virtual Reality Exergaming has a time-dependent relationship with the significant extension of one-legged standing time, and this high-level change shows good stability during the follow-up period. The initial improvement of balance ability under the condition of Traditional Sports Game Intervention was not significant, and the follow-up period was slightly improved but the range was limited. This result suggests that Immersive Virtual Reality Exergaming may provide an effective promotion path for autistic children balance and lower limb static control quality. However, limited by non-randomized design and the lack of treatment-free control group, the above findings should be used as exploratory tips, and the potential advantages of Immersive Virtual Reality Exergaming still need to be verified by more rigorously designed randomized controlled trials. The initial effect of Traditional Sports Game Intervention is not significant, but its auxiliary value in comprehensive rehabilitation cannot be ruled out.

## 4 Discussion

### 4.1 Principal findings

In many previous studies, the subjects often lack a control group, or the control group is a healthy child, so it is difficult to directly compare the effects of immersive virtual reality exergaming with traditional sports game intervention, and it is difficult to determine the specific efficacy of VR interventions for autistic children (39, 44). In this study, a cohort study design was used. All subjects were autistic children. The potential value of immersive virtual reality exergaming in improving motor ability and developmental functioning was preliminarily explored, and the immediate and delayed effects of VR somatosensory games and traditional somatosensory games were compared. The results showed that autistic children showed different degrees of positive trends in multi-dimensional indicators under the two intervention conditions. However, the advantages of VR intervention are not consistent among different indicators, and it is not appropriate to generalize the effect of VR intervention to all functional areas. In terms of gross motor, standing long jump, 20 m shuttle run, 4 × 10 m shuttle run, standing on one foot, language, personal social interaction and adaptability, the interaction effect between time and group reached a significant level (*p* < .05), suggesting that VR intervention has a time-dependent relationship with the significant improvement of these indicators. The improvement of the experimental group in the intervention period (T0 → T1) is greater than that of the control group. Among them, the interaction effect of standing long jump (F (2,76) = 40.669, *p* < .001) and 20 m shuttle run (F (2,76) = 15.908, *p* < .001, partial *η*^2^ = 0.295) is particularly prominent. During the follow-up period (T1 → T2), there was no statistically significant decrease in the score changes of Cohort 1 in the above indicators, indicating that the high-level change trend accompanied by immersive virtual reality exergaming has good stability. However, in terms of fine motor and grip strength indicators, the interaction effect of time and group did not reach a significant level (*p* = .496, *p* = .253). The two groups of change trajectories were basically parallel, and immersive virtual reality exergaming did not show additional gain. It is worth noting that although the above eight indicators showed the dominant trend of immersive virtual reality exergaming, most of the differences between the two groups at each time point did not reach a statistically significant level. The advantage of VR was mainly reflected in the increase in the magnitude of changes before and after the intervention in cohort 1, rather than the difference between the endpoint values. In addition, limited by the non-randomized design and the absence of a treatment-free control group, the observed changes may partially reflect the natural developmental maturity of autistic children, the practice effect of repeated tests, and the statistical regression effect. Therefore, the above findings should be interpreted as suggestive evidence, rather than the final conclusion of the long-term efficacy of immersive virtual reality exergaming.

The above results are consistent with some recent studies (36, 45), suggesting that VR technology may provide an effective promotion path for the improvement of developmental functioning and motor ability of autistic children through its immersive environment, multi-sensory integration and adaptive scene (46–48). However, the specificity, persistence and clinical significance of the effect need to be verified by more rigorously designed randomized controlled trials and larger sample studies. The heterogeneity in our findings,where VR demonstrated advantages in some domains but not others—is consistent with the broader literature. Malihi similarly reported that VR experiences in autistic children are highly individualized, with demographic and phenotypic characteristics moderating intervention outcomes (49). This supports the view that VR efficacy is context-dependent and should not be generalized across all functional domains (49).

#### 4.1.1 Immediate effects of VR-based exergames on autistic children's developmental functioning and motor skills

Based on the above results, we speculate that the dominant trend of immersive virtual reality exergaming may be partly attributed to the uniqueness of VR technology. Because autistic children have difficulties in information integration, it is difficult to effectively screen key information (50, 51). However, autistic children usually have the characteristic of visual precedence and strong visual memory ability (52). The rich virtual scenarios of the multimedia interactive system fit the cognitive characteristic of autistic children of “visual precedence” (44), providing a safe, controllable and visually immersive environment for autistic children (53). The immersion and controlled environment of VR not only enhance children's attention span but also help children show better attention in relevant tasks (54–56), enabling them to invest more energy in the VR classroom and achieve the effect of autonomous rehabilitation training. In addition, through the form of floor interactive games, the system combines VR tasks of multisensory stimulation to stimulate autistic children to engage in physical activities, effectively enhancing muscle strength and coordination. It is worth mentioning that the system has the function of reflecting children's movements in real-time on the multimedia screen or the ground space, significantly enhancing children's proprioceptive feedback. Thus, autistic children can observe their own movement performance in real-time during the interaction process, thereby obtaining positive reinforcement. This real-time feedback mechanism not only helps children perceive their own body movements more accurately but also further enhances sensorimotor coupling, effectively improving children's motor skills.

From the perspective of neural mechanism, previous studies have pointed out that the functional connection between the default mode network (DMN) and the dorsal attention network (DAN) in autistic children is relatively weak, and this connection strength is positively correlated with cognitive flexibility and social ability (57, 58).. The immersive environment of VR activates the prefrontal cortex and the posterior parietal cortex through dynamic visual and spatial audio cues, enhancing the functional connectivity between the DMN and the DAN, and improving the cognitive flexibility and environmental adaptability of autistic children in complex tasks (59, 60). However, it should be clearly pointed out that this study did not collect neuroimaging data, and the above mechanism speculation was only based on indirect evidence and literature inference, which could not be used as a confirmation of the effect of immersive virtual reality exergaming on specific brain networks. Compared with traditional sports game intervention, the latter mainly relies on repetitive movement training guided by the therapist, which may mainly activate local brain regions, and the degree of multisensory integration is relatively limited (61–63). This speculation is consistent with the trend of greater improvement in multiple exercise indicators in the experimental group in this study. From a behavioral perspective, the reward mechanism integrated into the system effectively improves the compliance of children, making them more willing to participate in the training. This is also consistent with our conclusion that the motor performance of Cohort 1 autistic children is better than that of Cohort 2. More importantly, the VR intervention based on this system also fully integrates modules such as cognitive training into sports games, combining them all to comprehensively promote the coordinated development of brain and physical functions.

#### 4.1.2 The sustainability of the intervention effect of immersive virtual reality exergaming on the development function and motor skills of autistic children

The preliminary results of this study showed that there was a time-dependent relationship between immersive virtual reality exergaming and the significant improvement of the development function and motor skills of autistic children in the intervention period (T0 → T1), and the change trend did not show a statistically significant decline in the follow-up period (T1 → T2), showing good stability. In contrast, the traditional sports game intervention had a relatively limited improvement in the score of the child development scale during the intervention period, and there was no significant regression during the follow-up period, but the overall level was maintained at a low level. In view of the fact that the change range of the two groups during the follow-up period did not reach a significant level, it is necessary to explain in particular that the core measure of sustainability advantage is not the change slope of the follow-up period, but the absolute end point level and the degree of lock-in of the previous advantage. Cohort 1 had established a significantly higher level of ability than the control group at the end of the intervention period (T1).

Taking personal social indicators as an example, the difference between the two groups reached 9.35 points at T1, and the difference was further stabilized at 11.95 points at T2 during the follow-up period. Although there was no significant regression in cohort 2, its stable absolute level was much lower than that in cohort 1. Therefore, the sustainability advantage of immersive virtual reality exergaming is reflected in the successful locking of high-level capabilities, rather than the fact that the follow-up period continues to rapidly widen the gap; the stable maintenance of cohort 2 at the low level does not have the clinical value of bridging the early gap. It is worth noting that the changes in the follow-up period of Gesell scale-related indicators (language, personal social interaction, adaptability) in this study are more gentle than those of sports ability indicators (such as standing long jump, 20 m shuttle run). This may be related to the calculation method of Gesell development quotient (DQ) -DQ = development age/actual age × 100. With the natural growth of children 's actual age, if the growth rate of development age fails to keep pace, the DQ score may tend to be flat. Therefore, the stable trend of Gesell related indicators during the follow-up period may be affected by age correction factors to a certain extent, while the exercise capacity indicators are absolute measurements and are not affected by this mechanism. However, the above explanation is only speculation, which needs further research to verify.

We speculate that children who receive immersive virtual reality exergaming are able to effectively transfer acquired skills into their daily lives, thereby maintaining or enhancing these skills through continuous practice (34). In addition, the high locking during the follow-up period associated with immersive virtual reality exergaming may be related to its technical characteristics. Because ASD individuals often show movement avoidance behavior due to sensory overload in the real environment, traditional sports game intervention mainly teaches skills in a controlled environment, but may not effectively reduce their anxiety level in the real environment, thus limiting the motivation of children to take the initiative to initiate sports in their daily lives. In contrast, the multimedia interactive system in the VR environment provides a variety of virtual scenes. Through the control of sensory input, it may help to reorganize the sensory processing mode and reduce the level of anxiety, thus promoting the participation of autistic children in sports tasks (44), And then reduce the adaptation of bad behavior (48, 64). Therefore, autistic children may feel less fear in the real environment and show stronger initiative in sports participation, thus creating conditions for skill generalization (65). Intensive practice in different VR scenes may help to transfer the acquired skills related to development functions to the real environment (66, 67)..However, the above-mentioned speculations on skill transfer and reduced anxiety levels are not the direct measurement results of this study, and need to be verified by further studies such as ecological validity assessment and daily behavior observation.

## 5 Conclusion

This study preliminarily suggests that immersive virtual reality exergaming shows a certain advantage trend in some developmental functions and motor skills indicators of autistic children, especially in language, personal social interaction, gross motor, standing long jump, 20 m shuttle run, 4 × 10 m shuttle run, one-legged standing and adaptability. There is a time-dependent relationship between immersive virtual reality exergaming and the significant improvement during the intervention period, and this advantage is stably locked during the follow-up period. Although the traditional sports game intervention also showed a certain improvement trend in some indicators, the range was relatively limited and the overall level was maintained at a low level. In addition, in terms of fine motion and grip strength indicators, the two sets of change trajectories are basically parallel, and immersive virtual reality exergaming does not show additional gain, indicating that its potential effects are not consistent in different functional areas.

Limited by the non-randomized design and the absence of a treatment-free control group, the above findings should be considered as exploratory tips rather than the final conclusion of long-term efficacy. The potential advantages of immersive virtual reality exergaming need to be verified by more rigorously designed randomized controlled trials.

### 5.1 Limitations and future research

The limitations of this study mainly lie in the subject scope and sample characteristics. Firstly, the study subjects were children with level 1–2 ASD aged 3–7. Although Chen et al. (68) indicated that young children might be unsuitable for VR training due to limited comprehension, this study found the VR system effective for them, which may be due to different VR modalities' applicability. However, to verify the generalizability of our findings across ASD populations, further studies on level 3 ASD children and younger or older age groups are still needed. Another limitation is the small sample size with male predominance. This aligns with ASD's higher male prevalence. While research shows no sex-based social skill differences, motor proficiency may still vary by sex. However, this study maintained a 100% retention rate of participants in the cohort analysis, without exit or loss of follow-up, avoiding the selection bias that may be caused by sample loss, and enhancing the internal and external authenticity of the research results. The underrepresentation of females remains a field-wide issue. Future research should give priority to recruiting female participants and increase the sample size to verify the universality of the results of this study in autistic children.

Our results highlight VR technology's significant potential. Future research should use more diverse standardized assessment tools, expand sample sizes, and integrate neuroimaging methods. Dynamically monitoring brain activation patterns via functional magnetic resonance imaging during intervention can deepen the exploration of VR intervention's neural regulatory mechanisms. This comprehensive approach can better investigate VR's impact on the social and motor aspects of ASD. In addition, more rigorously designed randomized controlled trials should be used in future studies, By optimizing research designs and enhancing theoretical foundations, we can provide stronger clinical support and clearer guidance for practitioners and VR designers (69). This will more effectively improve the life outcomes of children and adolescents with ASD, meet growing demands (8, 9), and promote VR technology's development in developmental disorder interventions.

## Data Availability

The original contributions presented in the study are included in the article/Supplementary Material, further inquiries can be directed to the corresponding author/s.
